# The Manchester Royal Lunatic Hospital
*Third Annual Report of the Medical Superintendent of the Manchester Royal Lunatic Hospital, situate near Cheadle, Cheshire, from June 25th, 1852, to June 24th, 1853. Manchester: Sowler. 1853.Second Annual Report of the Manchester Royal Lunatic Hospital, situate near Cheadle, Cheshire. In the year from June 25th, 1851, to June 24th, 1852. Manchester: Sowler. 1852.Report of the Manchester Royal Lunatic Hospital, situate in the Township of Stockport Etchells, near Cheadle, Cheshire. This Institution is in connexion with the Manchester Royal Infirmary. June, 1851. Manchester: Sowler.


**Published:** 1854-01-01

**Authors:** 


### Art. VII.—THE MANCHESTER ROYAL LUNATIC HOSPITAL.*

The "Third Annual Report of the Medical Superintendent of the Manchester Royal Lunatic Hospital" at Cheadle has just reached us. It demands special notice; but before proceeding to discharge a duty

* Third Annual Report of the Medical Superintendent of the Manchester Royal Lunatic Hospital, situate near Cheadle, Cheshire, from June 25th, 1852, to June 24th, 1853. Manchester: Sowler. 1853.

Second Annual Report of the Manchester Royal Lunatic Hospital, situate near Cheadle, Cheshire. In the year from June 25th, 1851, to June 24th, 1852. Manchester: Sowler. 1852.

Report of the Manchester Royal Lunatic Hospital, situate in the Township of Stockport Etchells, near Cheadle, Cheshire. This Institution is in connexion with the Manchester Royal Infirmary. June, 1851. Manchester: Sowler.

which we conceive devolves upon us, we shall briefly advert to the history of this establishment.

The Manchester Infirmary was opened in the year 1752, and the hospital for the reception of lunatics, being a separate foundation, was soon afterwards annexed to it. The original edifice, we need scarcely say, has undergone vast improvements; but its situation must still appear inconceivably bad. In the centre of this densely populated city, without any available area affording sufficient space for exercise or garden ground—with a sheet of stagnant water in the midst of a small space of blighted greensward, fenced round with iron railings, immediately before its façade, the stranger may well wonder that the "merchant princes" (as they were designated in the Netherlands during the palmy days of the Hanseatic league) of one of the most flourishing commercial towns in the world, should not have provided a more eligible locality for so important a public edifice. But as accidents are of frequent occurrence in the manufactories at Manchester, it may have been attended with serious inconvenience if the surgical hospital had been built at any distance from the town. The great hospitals of London, the Hôtel Dieu of Paris, are also in crowded districts; and so far, it is true, the position may be defended; but there can be no doubt that, for a lunatic asylum, the situation always must have been exceedingly objectionable. Independent of this, designed in the last century, we can well understand that its construction must have ill accorded with our present improved views of the accommodation required by the insane. The committee therefore determined upon detaching the lunatic hospital from the infirmary, and erecting an asylum in a more eligible situation. Accordingly they purchased a large piece of ground near the village of Cheadle, upon the Mersey, about ten miles from Manchester, and three from Stockport; and while the patients removed from the old building were temporarily distributed in surrounding asylums, the new edifice was steadily and rapidly proceeded with, and completed at the end of the year 1849. Here we may remark, and the history of the Manchester Infirmary in some measure justifies our opinion, that a lunatic asylum can never advantageously be made a *section* of a general medical hospital. The two cannot be combined under the same roof. A ward for the temporary treatment of the insane may be desirable in every hospital; but when the disease has progressed to a particular stage, *such cases can only be properly and efficiently treated in an establishment arranged for that special purpose.* In the December of 1849, the lunatic hospital at Cheadle was opened for the reception of patients; here, however, it may be necessary to explain that this institution does not come under the category either of a county or of a private asylum, but, founded upon charitable principles, it is in part only a private asylum; that is to say, it is an eleemosynary establishment for the reception of patients "whose circumstances are such as to render it undesirable to drive them to the disagreeable necessity of becoming inmates of the county lunatic hospital." (*Report* i. p. 4.) But there is this anomaly in its constitution—that while its benevolent fund is available for the support of patients who are unable to pay for their maintenance, it is also open for the reception of patients belonging to the highest class. "It is intended" (says the first Report) "for the reception of patients from various classes of society, commencing from the highest class, at weekly payments of 4*l*. 4*s*. and 5*l*. 5*s*., in proportion to the nature of the accommodation." (*Ibid.*) We have always contended that the two opposite classes of society, the rich and the poor, can never be domiciled with any degree of satisfaction or personal comfort under the same roof; for, strange as it may seem, there would appear to be as broad and as well defined a distinction between different classes of society, as between the different races of mankind, and their amalgamation would even appear to be as difficult. What is true as affects our social sympathies and habits in health, is equally true of them in disease; and the very equivocal success—we may almost, as will presently appear, venture to say the non-success of the Cheadle hospital as a remunerative institution, strongly corroborates the truth of our position.

Under these circumstances, the Manchester Royal Lunatic Hospital was transferred to Cheadle, and the first report of its committee appeared in June, 1851. One of the most prominent objects of this report is to intimate that the institution still continues in connexion with the Manchester Royal Infirmary, which is fairly enough described to the public as being one of its most attractive features. "Another important advantage," says the committee, "enjoyed by the institution, is its immediate and intimate connexion with the Manchester Royal Infirmary. The physicians of this last institution are also physicians of the lunatic hospital, and two of their number are chosen annually as visiting physicians for the year. The patients have thus secured to them the constant services of two of the most eminent and experienced members of the medical profession to advise with and to assist the resident medical superintendent." (*Report* i. p. 5.) We are, in a preceding paragraph, it should be observed, informed that another advantage "is to be found in the experience and efficient management of its resident medical superintendent, Mr. Dickson, ably assisted by his wife as matron, and by other members of his family who are resident in the institution." (*Ibid.*) Such, then, was the original medical organization of the lunatic hospital now removed to Cheadle; it consisted in the supervision of two visiting physicians belonging to the Manchester Infirmary, who were to be annually elected, and the personal superintendence of Mr. Dickson. Hence, to the first report of the committee we find annexed the "report of the medical officers," duly signed by Thomas Dickson, L.R.C.S. Edin., Resident Medical Superintendent, and countersigned by R. F. Ainsworth, M.D., and F. Renaud, M.D., Visiting Physicians for the year 1850-51. So far, so good; this was clearly enough all *en règle;*—but in the next, the second annual report for the year from June 25th, 1852, to June 24th, 1852, the committee of the institution set out with stating, that "instead of making any regular report of their own for the past year, they have thought it right to publish the report which they have received from the resident medical superintendent." (*Report* ii.) We have, then, a report drawn up by Mr. Dickson, which is *not* countersigned as before by the two visiting physicians. It is not for us to "sit by our fireside, and presume to know what is done i' the capitol," but we are left to surmise that the advantage promised to the institution of being placed under the supervision of two physicians, annually elected, had, at this early period of its history, for some reason or other been withdrawn; not that we mean to imply that the physicians belonging to the Manchester Infirmary are no longer connected with this establishment, for the reverse we know to be the case; nay, the chief business of the Cheadle Lunatic Hospital is, we believe, transacted in the committee-room of the Infirmary at Manchester. We point, however, to the significant fact of the two last annual reports not being countersigned by visiting physicians, with the view of exonerating any physician connected with the Manchester Infirmary from having had any share in the production, or even approving of, the report before us, which we have no hesitation in pronouncing unworthy of any public institution.

We are willing to make every allowance for Mr. Dickson's literary inexperience, and want of ability to express in classical language his ideas, for it is not every medical man who possesses the qualifications of a professed *littérateur;* but when he sat down to write his *third* report he ought to have had a clear conception of the duty which devolved upon him. Neither the public, nor, we presume, the physicians of the Manchester Infirmary, wished him to read them a lecture on insanity. He had an easier task—a simpler duty—to perform, which was, to give in plain unaffected language an "account of his stewardship" in connexion with the state and progress of the institution during the preceding year, accompanied by the usual statistical returns, and a brief description of any cases of special interest which may have come under treatment, interspersing the same with such clinical observations as any particular case might have suggested. The entire use and value of these reports must depend upon their conveying to us a faithful account of such matters as immediately concern the state and progress of the institution; wherefore the annual report of every lunatic asylum is *de facto* a chapter in its history which ought to be very carefully and circumstantially recorded; nor is it desirable that speculations should be intruded upon us which are wholly irrelevant to the current interest of such documents. We are, it is true, desirous of knowing the details of every important case which may occur in the Manchester Royal Lunatic Asylum; and we fully appreciate every statistical return connected with it, as entering into the elementary stream of a wide and general induction which may hereafter lead to important results; but we care not to see the medical superintendent of the hospital mount the *tripod* to deliver such oracles as are enumerated in the very first page of the report before us:

"*I* have, on a previous occasion," says Mr. Dickson, with an air of authority, "entered at some length on the question as to what constitutes insanity. *I* have there stated *my* views on the subject, and will at present merely quote in a sentence or two the opinions *I* have thus attempted to establish, by an induction of facts gathered from *my own* experience (!) and supported by that of others."

Here Mr. Dickson seems to exclaim with Hamlet—

"The time is out of joint; O cursed spite
That ever *I* was born to set it right."

But he quickly recovers himself, and over-exultingly thus explains his views:

"'Insanity is not a specific disease of itself, such as fever, gout, rheuma-
'tism, scrofula, consumption, &c., but is an evidence of disease or mal-
'formation existing in some part of the brain or of its membranes; this
'derangement giving rise to those mental symptoms which we call in-
'sanity.' Here *I* have asserted that insanity is not a *specific* disease; that it does not *operate* in the same manner as the *afore-mentioned* diseases; and that it is not to be classed among those which almost necessarily prove fatal, as consumption, &c., but is one of those most capable of successful treatment, and the most *durable* (?) of all diseases."

Upon what "previous occasion" Mr. Dickson delivered himself of this Shibboleth we do not care to inquire; the passage marked by inverted commas as a quotation does not occur in his two former reports; if, however, we do take the pains to unravel this rigmarole of very questionable and indifferent English, we shall find that if it convey any meaning at all, it is, that Mr. Dickson views insanity very much in the same light as other medical practitioners, excepting that he has a fancy to play upon the words "*specific disease*." We know of no medical writer who has contended that insanity is *par excellence* a *specific* disease, nor is it at all apparent what Mr. Dickson himself understands by the word *specific*; hence it would seem that he has set up a dogma of his own for the purpose of triumphantly knocking it down, and founding upon it the following very equivocal reasoning. "If, then," he continues, "insanity be not a specific disease, and if it be capable of such treatment as is likely to effect a cure, we can understand from observation of those who labour under it what is the nature of the disease, and what are the causes which have produced it." There is nothing very satisfactory in this style of argument. It does not follow that because a disease yields to medical treatment, that therefore its true pathology is unveiled to us. Neither is it by any means clear that the removal of the cause which has produced any particular form of mental disease would necessarily effect its cure.

"The producing cause," says Mr. Dickson, "being for the most part known (*query*) to all who have intercourse with the person affected, what does common sense say as to the remedy likely to prove most effective in the removal of the disease? The most obvious answer which suggests itself is this, 'Remove the cause and the effect will cease.' All other remedies must give way to this one; whatever prejudice under the garb of superior knowledge may suggest as to the cause or the treatment of the malady, must be rejected by every one who takes this common sense view of the matter, especially if by a sufficient induction of cases we can establish the fact that this is the best and most successful method of cure."

Every physician, as Mr. Dickson must be aware, recognises the old axiom, "Sublatâ causâ tollitur effectus;" and there can be no doubt that in the incipient stages of insanity, the removal of the exciting cause, whether moral or physical, may in many instances effect a cure; but unfortunately in a very large proportion of cases this wise precept will not hold good; the mind having received a sudden shock may, we all know, be shaken upon its throne and never again resume its empire. But what has this to do with the hospital at Cheadle, its state and progress during the past year, or with the official details which we might reasonably have expected to find in this report? Assuredly nothing! The two former reports of this institution by Mr. Dickson were business-like and to the point, but the subject-matter of the first half-sheet of the present, the third annual report, has no more to do with the Manchester Royal Lunatic Hospital than with the State Lunatic Asylum of Ohio, or with the proceedings which may, for aught we know, at this moment be going on in the camp of the great Cham of Tartary.

Unrelieved by a single ray of original thought, and expressed, to say the least, in very inelegant language, we are glad to get rid of these discursive introductory observations, and enter upon the proper business of the report, which appears to begin at the bottom of page 8, with a puffatory description of the hospital and its surrounding gardens.

We must here protest *in limine* against the annual reports of our lunatic asylums being converted into surreptitious advertisements; under the pretext of an official report, we occasionally find an ostentatious display palmed upon us of the advantages to be derived from this or that asylum, addressed indirectly to the public rather than to the governors of the institution or to the medical profession. This is obviously very much the character of the report before us; in proof of which we may subjoin the following detached passages, reminding the reader, upon the authority of Puff himself, that "puffs are of various sorts; the principal are the 'puff direct,' the 'puff preliminary,' the 'puff collateral,' the 'puff collusive,' and the 'puff oblique' or 'puff by implication.'"

"The hospital" (we quote the report before us) "is large and commodious, and fitted up with every convenience that can render it healthful and agreeable." (p. 8.) . . . "Opportunities for physical exercise are amply supplied by the farm and the gardens, comprising upwards of thirty acres." (*Ibid.*) . . . . "The hospital is amply supplied with all kinds of vegetables, fruits, &c., in their season, and several extensive flower-gardens." (p. 9.) . . . . "A very interesting part of the patients' work during last winter, was the transplanting of trees from those situations where, having been planted too closely at first, they had overgrown and become too thick for the purposes they had been designed to serve; these were transplanted from the avenues and gardens to form ornamental clumps in the field in front of the hospital." (*Ibid.*) . . . . "Two walks (in the kitchen garden) have been formed the entire length of the outside garden walls at the back of the hospital, intersected by cross walks from each of the back entrances; these walks are to be surrounded with a low fence and those decorations which will render them more pleasing and agreeable to the eye." (*Ibid.*) . . . "These operations have been entirely carried forward by the patients and their attendants, and it is delightfully interesting to see with what spirit and vigour they have been prosecuted." (*Ibid.*)

Here we have a very fair specimen of the meretricious style adopted by auctioneers in advertising estates for sale. No doubt the mind of Mr. Dickson is deeply imbued with this kind of classical literature; indeed, when he proceeds to describe more in detail the appointments of the hospital and management of the patients, the mantle of the late George Robins appears to be visibly descending upon his shoulders. He cannot mention the carpenter's shop connected with the hospital without reminding us that it "is fitted up with a turning-lathe (made by a patient), benches, and a variety of *such tools and other conveniences as may enable them to perform any such kind of work as is required.*" (p. 11.) The language, by the way, of the late George Robins, when upon this nether earth, was certainly more graceful than this. Nor can Mr. Dickson allude to the ordinary daily work executed by the patients without indulging in the same "Ercles vein;"—thus, "The summer-houses erected last year, and so much admired by the Commissioners in

Lunacy, all the outside gates and garden doors (twenty-one in number), and the iron fence in front of the hospital, have all been re-painted; in the interior, nine rooms have each received four coats of oil-paint, fourteen bedrooms, four sitting-rooms, and three galleries have been coloured, and the entire corridors of the servants' departments have been twice whitewashed." (p. 11.) Wonderful, most wonderful! Highly gratifying is it to hear that "throughout the whole of these operations the best and most cordial feelings have prevailed between the carpenter and the patients, and the most satisfactory results have followed to the patients' health." (*Ibid.*) Were details of this description set forth simply to show the governors of the hospital the system of discipline and management adopted in the establishment, it would be legitimate enough; but more than this is here attempted—the most common appointments in asylums, the most ordinary occupations and recreations of the patients, are surcharged in description for the obvious purpose of producing what in theatrical language is called "a striking effect." Take, for example, the following exaggerated account of a game of cricket:

"In connexion with these useful exercises," says Mr. Dickson, "may be classed such outdoor amusements as bowling and cricketing: in the latter recreation many of the patients have been greatly interested; an exercise so exciting in its nature, and so well fitted to call into play every muscle of the body, to excite every nerve, and also requiring such an amount of mental as well as physical activity, is well adapted to prove beneficial to the insane; occupied either in bowling or batting, the energies of their minds are called forth which had become dormant; and on these occasions, or when fielding out on all sides of the wickets, the emulation to catch the ball and displace the person occupying the much-envied position at the wickets was exciting enough to interest many of them intensely: from their own testimony, and from the evident delight which a looker-on could not fail to observe them to take in this exercise, it was evident that some of their happiest hours were passed in this exciting recreation." (pp. 11, 12.)

Bosh! It would evidently not have sufficed if Mr. Dickson had stated in his report that the patients had amused themselves occasionally by playing cricket, as they do at other asylums—there must be a coloured daguerreotyped picture, to produce effect: hence this ludicrous piece of clap-trap! So, too, he is not content with reporting that the rooms of the patients are provided with a certain number of books or magazines for their amusement; he must go further:

"Of periodicals," says he, "we receive 'Blackwood's Magazine,' 'Chambers's Journal,' 'Dickens's Household Words,' 'Bleak House,' 'Illustrated London News,' 'Punch,' &c. Of newspapers, we get the 'London Times,' 'The Manchester Examiner and Courier,' 'Stockport Advertiser,' 'Ladies' Journal,' &c. Some of the patients are keen politicians, and pay great attention to every political movement that is taking place in the world: the amount of intelligence they manifest and the evident delight they derive from information as to passing events, is strikingly manifest, and proves that though their minds may be erratic and adrift on some points, on others they reason and judge with as much correctness as if they were perfectly sane; with such persons the reading-room is a favourite retreat." (p. 12.)

The fact here referred to must be familiar enough to men who have ever had the charge of a lunatic asylum, but why so much overcolouring of detail? It may please Mr. Dickson to dwell upon the healthful and agreeable locality of the hospital, the completeness of its internal arrangements, the comforts provided for the patients; but what, after all, is the true state of the case? The Commissioners in Lunacy, before approving of the erection of an asylum, or granting any house a licence, require that it shall be situated in an open, cheerful, salubrious locality; hence the asylums of England are for the most part to be found in the most beautiful spots that can be selected; they also make it imperative that the house shall stand on a certain amount of acreage, for gardening or agricultural purposes, and that airing courts and pleasure-grounds shall be accessible to the patients; they furthermore insist upon the apartments, whether public or private, being comfortably and even handsomely furnished, and direct that the patients shall be supplied with the means of occupying and amusing themselves with books, games, &c. But no well-conducted asylum would ever dream of advertising these as special attributes—they are the common conditions which the Commissioners in Lunacy require shall be provided in every asylum.

One of the evils arising from Mr. Dickson's hyperbolical style of diction is, that a false colouring is reflected upon the actual state of the institution; thus, he sets out with announcing that "never before has this institution better answered the purposes for which it has been erected, or more fully met the anticipations of its projectors." Why, the hospital has not been opened more than four years, although the foundation of the institution may carry us back to the last century. And what has been the success of which Mr. Dickson boasts? We may form some idea by the following tabular view, constructed from the statistical tables in the three reports before us, exhibiting the state and progress of the hospital since it opened.

| | Admitted. | Discharged Cured. | Discharged Relieved. | Died. | Remaining in Hospital. |
|---|---|---|---|---|---|
| From the opening, December, 1849, to June, 1851 . . | 65 | 17 | 7 | 6 | 33 |
| From June, 1851, to June, 1852 . . | 33 | 17 | 4 | 8 | 37 |
| From June, 1852, to June, 1853 . . | 35 | 16 | 5 | 4 | 47 |

We confess that the table before us does not suggest any particular cause for congratulation : look at the admission column ; between 1851 and 1852, there were 33 admissions ; and between 1852 and 1853, only 35 admissions. Then, when we refer to the Commissioners in Lunacy's annual reports, we find that on the 1st January, 1851, there were 23 patients in the hospital, and on the 1st January, 1852, only 33. Yet is this hospital constructed for the reception of 100 inmates, situated in the centre of one of the most populous manufacturing districts in England, having all the interest at its command which the Manchester Royal Infirmary and its eminent physicians can bring to bear upon it. We believe that the institution is in every respect well conducted; and although we see every reason to condemn unsparingly the report before us, we believe that Mr. Dickson, in his capacity as medical superintendent, discharges his duties ably, and is entitled to the highest confidence ; but, as we above hinted, the success of the hospital is obviously so very equivocal as not to warrant its third annual report opening with a flourish of trumpets. There may be many reasons for the admissions at Cheadle Asylum being fewer than might have been expected; nor does the circumstance militate in any way against the management of the establishment. Since the opening of the Rainhill Asylum, ample provision has been made for the lunatic poor of Lancashire, which has now three very large and admirably organized asylums at its command. Near Manchester and Liverpool, it has also to contend with the competition of five private asylums. And here we may venture to relate an anecdote : when we were recently in Manchester, we were speaking to a physician concerning the hospital at Cheadle, when he mentioned that he had met with several patients he was desirous of sending there, but he could not prevail on the relations or friends to do so, because they entertained a notion that, being in connexion with the Manchester Infirmary, the institution was a public charity. Here, then, we meet with an additional illustration of the predominance of that feeling—call it an incurable prejudice if you will—which we have so often pointed out as throwing an insuperable barrier between private and public asylums.

To return to the report before us. We find Mr. Dickson vaunting in no measured terms of the success which has attended his treatment of recent cases of insanity; but figures, like edged tools, are dangerous weapons, and when we compare the statistics he has laid before us in the second and third reports together, we again find no very great cause for self-congratulation. The average number of patients admitted during the past year, says the report of 1852, was 36. Of the patients discharged, 17 were cured; being in the ratio of 51·22 per cent. to the number of admissions, or of 47·22 per cent. to the average number resident. Then turning to the third report, for

1853, we read—"The average number daily resident throughout the year was 39·53. Of the patients discharged, 16 were cured, being in the ratio of 42·8 per cent. to the number admitted, or of 41 per cent. on the average number resident." The curative results, therefore, exhibited in the third annual report, are less satisfactory than those in the second, there being a falling off in the percentage of cures upon the number of patients admitted of 9·14 per cent., and on the number of resident patients, 6 per cent. We do not refer to this less favourable return of cures with any ungenerous views: the falling off doubtless occurred from circumstances over which Mr. Dickson had no control; but, with his statistical tables before him, he might have spoken in that tone of moderation which becomes every medical man who knows how precarious, from year to year, are the results, in all diseases, of the best medical treatment.

The most objectionable, or rather the most reprehensible, portion of this report is its conclusion, where we find that Mr. Dickson has adopted a course which we believe to be unprecedented in the drawing up of these annual reports. After having lauded to the skies the Royal Hospital, under his immediate superintendence, and given the most flattering account of every department connected with it, he concludes his report by annexing to it a series of eulogistic memoranda which his personal friends who have visited the hospital have, strange to say, been permitted to inscribe in the "Visitors' Book." He first lays before us a copy of an entry by the Commissioners in Lunacy, dated the 22nd September, 1852, in which they state: "We think that the general condition of the hospital and patients is creditable to Dr. and Mrs. Dickson, and we only have to regret that so few patients have the benefit of the good accommodation provided." This must have been very gratifying to the superintendent, but it is by no means clear to us that the commissioners intended their entry for publication: be this as it may, the Earl of Shaftesbury, in November last, visited Manchester on one of those philanthropic occasions which he so frequently and so generously attends, identifying his noble name—illustrious in the annals of literature—with every good and charitable undertaking which promises to ameliorate the social and religious condition of the working classes—when, of course, the opportunity was not lost sight of by Mr. Dickson, of inducing his Lordship to visit the Royal Hospital at Cheadle. We have, therefore, annexed to the entry of the commissioners, a memorandum from the noble Earl—stating, as Chairman of the Board of Lunacy—that he fully concurs in the last report of his colleagues.

Here we may observe that we have very great doubts as to the propriety of publishing the entries which the Commissioners in Lunacy make at their official visits without their special sanction, which would even then have the effect of creating an invidious distinction between asylums which may be all equally well conducted. Such entries are made by the Commissioners in Lunacy as administrators of the law, and in accordance with the directions of the Act of Parliament. They ought not, we apprehend, therefore to be paraded forth as advertisements, to serve the private interest either of individuals or institutions; but had Mr. Dickson stopped here, whatever we might have thought of his taste or discretion, we should not have accused him of any very marked transgression. He has, however, gone much further than this; he has induced unofficial persons, who happened to be passing through the neighbourhood—besides his personal friends—to visit the hospital at Cheadle, and then record their favourable impressions; and in this manner has he collected a series of eulogistic demonstrations, which are published at the end of the report before us as being "Extracts from the Visitors' Book." We did not, for our own parts, require Mr. Dickson to call "witnesses as to character;" and we have no hesitation in saying, that in doing this, he has had recourse to a proceeding which is wrong in principle and vicious in tendency. But before referring more particularly to these complimentary effusions, we should like to inquire what right any unofficial persons have to inscribe any observations whatever in the "Visitors' Book?"

The Act of Parliament (8 and 9 Vict., cap. 100, sect. 65) enacts that this "Visitors' Book" shall be kept in every licensed house and in every hospital in which lunatics are received, for the Commissioners in Lunacy and Visitors respectively to enter therein at the time of their visitations the results of their inspections and inquiries, with such observations as they may think proper to make. It is therefore the book appointed by law to be kept as the official record of every licensed house or lunatic hospital; as such it has always been set apart and esteemed by us, and we confess we never before heard of its being handed about from stranger to stranger by any medical superintendent, for the purpose of getting their "favourable impressions" inscribed in it. The testimonial system we believe to be thoroughly rotten at the core; and we confess we are sorry to see this attempt made to introduce it into our annual lunacy reports. Here we have first, as an extract from the "Visitors' Book," a complimentary effusion from Mr. Powell, inspector of asylums, Mauritius. He states—"I have spent some days with Mr. Dickson, and feel unwilling to leave without some record of the very favourable impressions left on my mind from what I have observed of the manner in which this institution is conducted by Mr. and Mrs. Dickson." No doubt Mr. and Mrs. Dickson are very good, kind, hospitable people, and their courtesy well deserved some requital; but what business the inspector of asylums at the Mauritius had to insert his grateful impressions in the "Visitors' Book" of the asylum we are at a loss to discover. The fashion, we believe, still prevails at the first-class hotels at watering-places for a "Visitors' Book" to be presented to the passing traveller when he pays his bill, that he may enter in it before he takes his departure some little laudatory acknowledgment of the excellence of the fare, the civility of the attendants, and the comfort of the accommodation; the same plan seems to be adopted by Mr. Dickson at the Royal Hospital, and it is evident that the inspector of asylums, Mauritius, could not, with a good grace, have done less on leaving the Institution than give Mr. Dickson the eulogium before us. Next comes a gratifying memorandum from Dr. Williams, Physician to the North Wales Lunatic Asylum; he, too, has of course great pleasure in recording his "favourable impressions" of his visit to the Royal Asylum at Cheadle—"very creditable, indeed, to Mr. and Mrs. Dickson;" "air of comfort and cleanliness" about the asylum, "which cannot be surpassed;" "patients happy;" what more can be desired? Next follows another highly satisfactory extract from the "Visitors' Book," by Dr. Kingdon, the Superintendent of the Lunatic Hospital at Exeter; he likewise has no hesitation in expressing his unqualified admiration of all that he saw and heard at the Royal Hospital. Other testimonials in the same panegyrical strain follow, among which we observe one from Dr. Burton of Maryborough, another from Professor Anderson of Glasgow, another from Dr. Hubertz of Copenhagen; but let Mr. Dickson for a moment pause, and ask himself what is the real, the intrinsic value of these eulogia? The simple fact of their being printed at the end of this report shows clearly that, whether solicited or volunteered, they were written with that view, but instead of creating a favourable impression on our mind they have the very contrary effect, inasmuch as they suggest to us that Mr. Dickson must have felt conscious that he needed such support to give *éclat* to his annual report, which presents to us the type of every feature which should be avoided in drawing up such a document. We have said that the tendency of the plan which Mr. Dickson has adopted in annexing these flattering "extracts" from the "Visitors' Book" to his report is vicious; we repeat it, for if the superintendents of other asylums did the same, where would it end? An unworthy competition in advertising themselves would be provoked, which would be derogatory to every institution which followed Mr. Dickson's example. How easy to seize an "individual of the highest eminence in this department of medical science" by the button and invite him to dine with us; how pleasant to discuss the chances of the war between Russia and Turkey over a bottle of old port wine before a glowing and cheerful fire; how agreeably passes the evening until the hour comes when our "eminent" friend must perforce take his departure, and will he do so without leaving behind him some slight record of the favourable impression which we hope to have made upon him? "Not for the world!" "Shall we ring the bell?" "John! bring the 'Visitors' Book!" "Infinitely obliged—very kind of you!" "It shall be published in my next annual report!"

---

### Art. VIII.—PROFESSOR VALENTIN'S PHYSIOLOGY.*

